# In vitro efficacy of vancomycin combined with fosfomycin against Vancomycin-Resistant Enterococci strains

**DOI:** 10.12669/pjms.36.2.1347

**Published:** 2020

**Authors:** Gulseren Aktas

**Affiliations:** Gulseren Aktas Ph.D. Department of Medical Microbiology, Faculty of Medicine, Istanbul University, Istanbul-Turkey

**Keywords:** Vancomycin, fosfomycin, combination, vancomycin-resistant enterococci

## Abstract

**Objective:**

Enterococci have been isolated frequently worldwide and have difficulties in the treatment. Combination antibiotherapies have a distinct advantage over monotherapies in terms of their synergistic effect. In the study, it was aimed to investigate in vitro activity of vancomycin combined with fosfomycin against VRE strains.

**Methods:**

A total of 30 VRE strains were included in the study. Bacterial identifications of the strains were undertaken using conventional routine methods. The resistance to agents tested was investigated by using the broth microdilution method. Glucose-6-phosphate (25 mcg/mL) for fosfomycin were used in all experiments. The activity of antibiotics in combination was assessed using a broth microcheckerboard. The fractional inhibitory concentration index (FICI) was interpreted as follows: synergism, FICI ≤0.5. Additionally, two strains in 30 VRE were studied to determine the time-kill curves to verify the synergistic results. For each strain, antibiotics were studied alone and in combination at the minimum inhibitory concentration (1xMIC) values.

**Results:**

Susceptibility rate to fosfomycin was found at 26.6 % (8/30). The MIC_50_, MIC_90_ and MIC interval values of antimicrobials were 512, 512, and 512 - 1024 mcg/mL for vancomycin, and 128, 160, and 64 - 224 mcg/mL for fosfomycin, respectively. The rate of synergism was found as 100 % by both checkerboard and time-kill methods.

**Conclusion:**

The result shows that the combination of vancomycin with fosfomycin could be an alternative in the treatment of infections caused by VRE.

## INTRODUCTION

Enterococci cause serious infections in society, and in hospitalised patients.^1^ In addition, entorocci are the problematic bacteria due to their natural resistance to some of the frequently used antibiotics and due to antibiotic resistance as a consequence of genetic material inheritance or mutations.^2^,^3^

Vancomycin-resistant enterococci associated infections have been increasing in recent years despite the coming into use of the new antimicrobials in the treatment of infections caused by the pathogen bacteria with multiple antibiotic resistance. The number of antimicrobial agents to be used in treatment is limited. Fosfomycin inhibits the cell wall synthesis by irreversibly binding to an enzyme (MurA; UDP-N-acetyl glucosamine-enolpiruvil transferees) that is responsible for the formation of UDP-N acetylmuramic acid in the first phases of peptidoglycan synthesis in the bacteria.^4^ This mechanism of fosfomycin makes the probable cross-resistance (almost) impossible with other antimicrobials and provides the possibility of the combined use of many antimicrobials.^5^,^6^ In addition, fosfomycin is an antibiotic which is worth investigating because it has single dose superiority, wide effect spectrum, little toxic adverse effects, and a longer half-life.^7^

One other option that can be used in the treatment of the infections caused by resistant bacteria is the use of the combination of the antimicrobial substances. Combination treatments increase the possibility of treatment owing to their wide effect spectrum and synergistic effect compared with the single medication treatment. In addition, the rapid increase in the rates of drug resistance in bacteria worldwide, and the inadequacy in the development of new antibiotics necessitated the formation and development of combined use of antibiotics studies.^8^ Therefore, in this retrospective study, the effect of the combination of vancomycin and fosfomycin in the treatment of vancomycin-resistant enterococci strains were investigated in *in vitro* environments.

## METHODS

A total of 30 VRE strains had been isolated from 16 urines, and 14 blood cultures which were randomly selected from different patients who had been presented to the various clinics of the university hospital were used in the study. Strains isolated from routine materials have been protected at -70°C deepfreeze in Brain-Hearth Infusyon broth (Oxoid, England) conteining 20% glycerol. When study were designed, they took out from deepfreeze and subcultured two times on TSA (Oxoid, England) to make them refresh, then, these strains were used in the study, retrospectively. Conventional routine methods were used in the identification of the strains. The Strains were identified as *Enterococcus* genus if they had the following properties: Gram-positive; catalase negative; ability to grow in 6.5% sodium chloride, 40% bile, and hydrolyzed esculin; and positive results of pyrrolydonyl arylamidase tests (PYR; BD: USA). In addition, the *Enterococcus* species were identified by investigating of movement, pigment formation, and biochemical features which were searched by both in-house and commercial identification for enterococci (Microgen Strep ID, Microgen Bio products, UK).^9^

Since Vancomycin resistance was the criteria for the study design, all *Enterococcus* spp. strains were investigated for vancomycin resistance. It was detected using the disc diffusion method (vancomycin; 30 mcg, Oxoid, England) first, and then, the results were confirmed using the broth micro dilution method.

Vancomycin (vancomycin; Multicell, USA), and fosfomycin (Bilim Ilac A.S., Istanbul) were used as the antimicrobial materials in the study. The minimum inhibitory concentration (MIC) values of both vancomycin and fosfomycin against 30 VRE strains was investigated using the broth microdilution method in accordance with the recommendations of the Clinical and Laboratory Standards Institute (CLSI), and the manufacturing companies.^10^,^11^ Cation-adjusted Mueller-Hinton II broth (CAMHB) (BBL™, Becton, Dickinson and Company, France) was used in the tests. In addition, glucose-6-phosphate (25 mkg/mL) (Sigma Aldrich Chemie Gmbh., Germany) was added in testing fosfomycin. The inoculum of each strain for the test was adjusted to achieve a final inoculum of 10^5^-10^6^ cfu/mL in the wells of microplates. The MIC values of antimicrobials were identified as the lowest concentration where bacterial reproduction could not be observed with the naked eye after 24 h incubation at 35°C. The results were evaluated in accordance with the CLSI criteria. Five VRE strains that were found resistant to fosfomycin (resistance criteria: MIC≥256 mcg/mL) in the first evaluation, then, were studied again in 160, 192, 224 mcg/mL interval fosfomycin concentrations. Additionally, these strains were tested by epsilometer tests (e-test) for fosfomycin (FOS, Liofilchem® s.r.l., Italy) by using Mueller Hinton II Agar added glucose-6-phosphate. The quality-control testing procedures were performed by using *Enterococcus faecalis* ATCC 29212 as reference strains in each run of broth microdilution test.

The in vitro activity of vancomycin and fosfomycin combination was identified using the broth microcheckerboard dilution technique.^12^ The concentrations of antibiotics in combinations were based on two dilutions above the MICs and four dilutions below. The final inoculum of the strains was approximately 10^5^-10^6^ cfu/mL in the microplate wells. The MIC values of each antibiotic in the combination were identified as the lowest concentration where the reproduction has ended visually after incubation for 24 hours at 35°C. The fractional inhibitory concentration (FIC) indexes (FICI) were calculated for each combination using the equation, FICA + FICB = FICI, where FICA is the MIC of drug A in combination divided by the MIC of drug A alone, and FICB is the MIC of drug B in combination divided by the MIC of drug B alone. Synergism was indicated by FICI ≤ 0.5.^13^

The time-kill curve method was performed in a randomly selected two VR-*E. faecium* strains to verify the synergistic results of the combination against VRE strains.^12^ In the test, antibiotics were studied alone and in combination at the 1xMIC concentration for each VRE strain. An antibiotic-free control was included as growth control.

An inoculum was produced by diluting the culture in CAMHB, which was obtained by overnight culture of the strain incubated in a calibrated shaking water bath at 70 cycles/minute at 35°C (GFL – 1092, Kisker Biotech GmbH and Co. KG, Germany). The inoculum was added to all flasks to yield a final concentration of approximately 1x10^6^ cfu/mL (Eliopoulos, G.M., 2005). The flasks which had a final volume of 10 mL, was continued to incubate in the same conditions. The viable counts were determined at intervals of 0, 4, 8, 12, 24, and 36 h after inoculation by sub culturing 0.1 ml from each repetitive serial dilution to 10^-7^ in Eppendorf tubes containing sterile saline (0.9% NaCl). The subcultures were done on plates containing Tryptic Soy Agar (TSA) (Difco™, Becton, Dickinson and Company, France) and were incubated at 35°C for 24 hour. The developed colonies were calculated, and the bacterial intensities in each bottle were counted. The synergistic effect of the combination was identified at a 100-fold increase in killing at 24 hours (as measured by colony count) or more with the combination, in comporison with the most active single antibiotic.^12^

## RESULTS

Of the 30 clinical VRE strains, 28 were identified as *E. faecium*, and two strains were as *E.faecalis*. In accordance with the obtained MIC values with broth microdilution, all VRE strains were found resistant to vancomycin, and 8 strains (26.6%) were found sensitive to fosfomycin because the fosfomycin MIC values were found as MIC ≤ 64 mcg/mL. The MIC_50_, MIC _90_ and MIC _interval_ values of the antimicrobials were found as 512, 512 and 512-1024 for vancomycin; and as 128, 160 and 64-224 mcg/mL for fosfomycin, respectively. Five strains were found resistant to fosfomycin (MIC: 256 mcg/mL) in the first evaluation. After additional microdilution study of fosfomycin for the five fosfomycin resistant strains were done, the MIC values were found as MIC: 160 in two strains, as MIC: 192 in two strains, and as MIC: 224 mcg/mL in one strain. As a result, 8 strains (26.6%) were found sensitive to fosfomycin (MIC≤64 mcg/mL), and the MIC _50_, MIC _90_ and MIC _interval_ values of 30 strains were found as 128, 160 and 64 – 224 mcg/mL, respectively. Twenty two strains (73.4%) were evaluated as less sensitive. No resistant strain was detected. Additionally, the result of these five resistant strains were found as the MIC: 196 mcg/mL by e-test.

The combination of vancomycin and fosfomycin studied for 30 VRE strains with checkerboard test was found to have a synergistic effect in all strains (100%). The distribution of the FICI values is demonstrated in Table-I.

**Table-I:** The distribution of FICI values of the combination against 30 VRE strains.

Combination	Distribution of FICI values (n)					Synergism % (n)

	0.1	0.2	0.3	0.4	>0.5	
VAN + FOS	8	16	5	1	-	100 (30)

**Fig.1:**
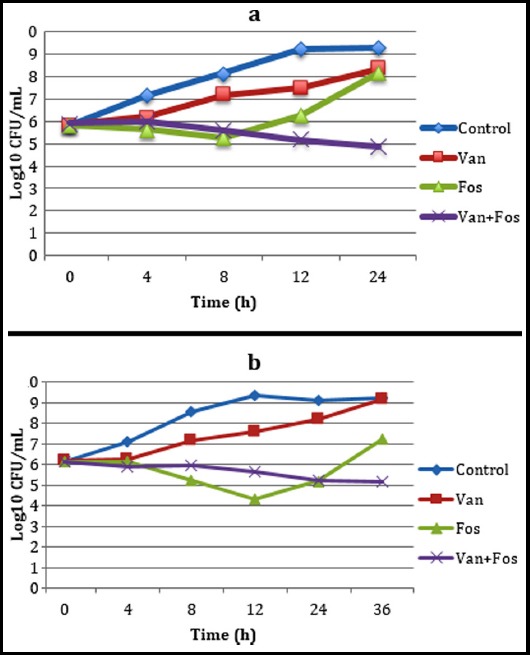
a, b Time-kill curves obtained with combinations of vancomycin and fosfomycin against two VRE strains.

The MIC_50_, MIC_90_ and MIC _interval_ values of each antibiotic alone in the combination were found as 32, 32 and 16-32 for vancomycin, and 16, 32 and 8-32 mcg/mL for fosfomycin, respectively. All strains were found resistant to vancomycin, and sensitive to fosfomycin in accordance with these concentration values. In addition, two randomly selected VR-*E. faecium* strains were also studied using the time-kill method to confirm the results. Their results were found to be synergistic at the 1xMIC concentration of antimicrobials against two *E. faecium* strains (Fig. 1 and 2). Synergy was defined at 24 h in strain 1 (Fig.1) and 36 h in strain 2 (Fig.2). No bactericidal effect of the combination was detected in two VRE strains studied using the time-kill at the end of 36 hours. The comparative results of dilution, checkerboard, and time-kill studies against two VRE strains were shown in Table-II.

**Table-II:** The comparative results of dilution, checkerboard, and time-kill studies against the two VRE strains.

VRE No.	MIC results (mcg/mL)			FICI	
	
	Dilution		Checkerboard*		Time Kill Results*
	
	MIC_VAN_	MIC_FOS_	MIC_VAN/FOS_		Synergistic Effect**
1	512	128	32/16	0.2	+
2	512	160	32/16	0.2	+

* Concentrations of both antimicrobials were at 1xMIC for each strain in the tests;

** ≥2 Log_10_ cfu/mL reduction.

## DISCUSSION

Fosfomycin was obtained from *Streptomyces fradiae* (ATCC 21096) first in 1969 by two pharmacist companies in Spain and came into use by 1970s.^14^ It is a wide-spectrum, natural phosphonic acid derivative antibiotic with bactericidal effect.^15^ Fosfomycin is an antibiotic that is used in the treatment of non-complicated urinary system infections. Currently, there has been an increase in the combined use of fosfomycin in the treatment of infections occurring due to the multiple antibiotic resistance of bacteria because the antimicrobial resistance increased, and there are difficulties to develop a new antimicrobial drug.^5^,^15^-^17^

The sensitivity of fosfomycin was detected as 26.6% (8/30) in the study. But, it was detected as 100% (30/30) in the combination alone. The obtained MIC values of vancomycin alone in the combinations were within the concentration values that could reach in human body.^7^,^18^ This suggests that the studied combination might be useful in treatment.

In a similar study conducted in Greece, fosfomycin was studied using disc diffusion method against a total of 166 enterococci strains consisting of 115 *E. faecalis* (7 strains VRE) and 51 *E.faecium* (19 strains VRE), and all strains were found resistant.^19^ In another study, fosfomycin MIC values against a total of 19 VRE strains consisting of 10 strains of VR-*E*. *faecium, and* 9 strains VR-*E. faecalis* were investigated using the agar dilution method, and MIC_50,90_ values were detected as 128 mcg/mL. The sensitivity for fosfomycin was reported as 30% in VR-*E*. *faecium* strains, and as 44% in VR-*E. faecalis* strains.^20^ In the same study, the synergistic interaction of vancomycin and fosfomycin was investigated using the time-kill method, and the synergistic effect were reported as 30% and 33%, respectively.

Although some standards recommended the agar dilution method that is conducted using the 25 mcg/mL glucose 6 phosphate included Mueller-Hinton agar/broth for *E. faecalis* strains isolated from urinary tract infections in vitro sensitivity studies with fosfomycin^11^, both agar dilution and broth dilution methods are used in the detection of the in vitro sensitivity of gram positive and gram negative bacteria to fosfomycin.^17^,^21^-^24^

Owing to the increase of drug resistance in pathogen microorganisms, and the inadequacy in the development of new antibiotics, there has been an increase in the use of combined treatment of fosfomycin for infections of the multiple resistant bacteria.^5^,^8^,^16^,^17^ This study is the first vancomycin and fosfomycin combination study against clinical VR-*E. faecium, and* VR-*E*. faecalis strains in Turkey. The combination was found to have synergistic effect in the total of 30 VRE strains (100%), both sensitive to fosfomycin (MIC ≤ 64 mcg/mL) and less sensitive to fosfomycin (MIC> 64 – <256 mcg/mL). The reason for the synergistic effect is unknown. In similar studies, the combination of fosfomycin and daptomycin was investigated against the VRE strains sensitive/less sensitive to fosfomycin, the reason for the detected synergistic effect was explained to be due to that fosfomycin caused a reduction in the surface load of the cell with the entrance of fosfomycin into glucose 6 phosphate, thus binding of the other antibiotic to the cell increased, and finally synergistic effect developed.^5^,^16^,^17^ The reason for the synergistic effect of fosfomycin and vancomyin combination in VRE strains may be theoretically explained with the results of this study that vancomycin inhibits the cell wall synthesis and also nonspecifically binds to the cell wall. Then, it affects the cell autolysins, and may cause the physiologic hydrolysis of the peptiglycan. Vancomycin also affects the RNA synthesis.^18^

## CONCLUSIONS

In vitro activity of vancomycin combined with fosfomycin against clinical VRE strains were investigated and it has been seen that the antimicrobial efficacy increased in the combined use of fosfomycin and vancomycin and no antagonism seemed. The results obtained from the study suggest that vancomycin and fosfomycin might be a new alternative to the limited number of treatment options of the VRE infections if more *in vitro* experiments and *in vivo* applications on this combination are proven.
